# Assessment of Negative Symptoms in Schizophrenia: From the Consensus Conference-Derived Scales to Remote Digital Phenotyping

**DOI:** 10.3390/brainsci15010083

**Published:** 2025-01-17

**Authors:** Armida Mucci, Stefan Leucht, Giulia M. Giordano, Luigi Giuliani, Sophia Wehr, Lucia Weigel, Silvana Galderisi

**Affiliations:** 1 Department of Mental and Physical Health and Preventive Medicine, School of Medicine, University of Campania Luigi Vanvitelli, Largo Madonna delle Grazie 1, 80135 Naples, Italy; 2 Department of Psychiatry and Psychotherapy, School of Medicine, Technical University of Munich, Klinikum Rechts der Isar, Ismaningerstrasse 22, 81675 Munich, Germany

**Keywords:** negative symptoms, BNSS, CAINS, digital phenotyping, EMA

## Abstract

The assessment of negative symptoms in schizophrenia has advanced since the 2006 NIMH-MATRICS Consensus Statement, leading to the development of second-generation rating scales like the Brief Negative Symptom Scale and the Clinical Assessment Interview for Negative Symptoms. These scales address the limitations of first-generation tools, such as the inclusion of aspects that are not negative symptoms and the lack of assessment of the subject’s internal experience. However, psychometric validation of these scales is still in progress, and they are not yet recommended by regulatory agencies, thus limiting their use in clinical trials and settings. Complementing these traditional methods, remote digital phenotyping offers a novel approach by leveraging smartphones and wearable technology to capture real-time, high-resolution clinical data. Despite the potential to overcome traditional assessment barriers, challenges remain in aligning these digital measures with clinical ratings and ensuring data security. Equally important is patient acceptance, as the success of remote digital phenotyping relies on the willingness of patients to use these technologies. This review provides a critical overview of both second-generation scales and remote digital phenotyping for assessing negative symptoms, highlighting future research needs.

## 1. Introduction

Negative symptoms refer to impairments or reductions in typical functions and behaviors related to motivation, interests, and emotional processes [1,2,3,4]. From the earliest descriptions of schizophrenia, these symptoms have been recognized as a core dimension of the disorder [5,6], significantly impacting patients’ functional outcomes, quality of life, and long-term prognosis [3,7,8,9,10,11]. Historically, the assessment of these symptoms—which include a loss of motivation and reduced emotional expression—has been inconsistent due to the lack of a consensus definition and the limitations of traditional scales, such as the Positive and Negative Syndrome Scale (PANSS) and the Scale for the Assessment of Negative Symptoms (SANS) [3]. These challenges in establishing standardized criteria have hindered the accurate identification of the complex clinical presentation of negative symptoms, complicating their effective management and treatment [3,12].

In response to these challenges, in 2005, Stephen Marder, Wayne Fenton, William T. Carpenter, Jr., and Brian Kirkpatrick organized a consensus development conference at the NIMH Neuroscience Center in Rockville, Maryland [13]. The primary goals of this conference were to establish a clear and unified definition of the negative symptom construct and to identify universally accepted, evidence-based measures and methodologies for testing the effectiveness of treatments targeting these symptoms. The conference identified five key domains of negative symptoms: blunted affect (a reduction in emotional expression, including facial, vocal, and bodily cues), alogia (a reduction in the quantity of spoken words and spontaneous elaboration), asociality (reduced social interactions driven by a diminished motivation to form and maintain relationships), anhedonia (a decreased ability to experience pleasure, distinguishing between pleasure during activities—consummatory anhedonia—and anticipated pleasure for future activities—anticipatory anhedonia), and avolition (a lack of motivation leading to reduced initiation and persistence in goal-directed activities) [3,13]. These symptoms present a certain degree of intercorrelations, forming two major factors: the Motivational Deficit (MAP), including avolition, anhedonia, and asociality, and the Expressive Deficit (EXP), comprising blunted affect and alogia. The two-factor model is supported by evidence of distinct behavioral, neurobiological, and clinical correlates for each of the two factors [1,2,3,14]. However, more recent studies, using confirmatory factor analysis, have suggested that a five-factor model (where each negative symptom domain represents a factor) or a hierarchical model (with the five negative symptoms as first-order factors and the two broader domains—MAP and EXP—as second-order factors), better fit the data, irrespective of the assessment scale, sample nationality, and stage of illness [3,15,16,17,18,19,20].

During the Consensus Conference, the experts recommended the development of new tools to assess the five negative symptom domains. The commonly used scales, SANS and PANSS, were found to include elements that do not belong to negative symptoms, fail to differentiate between anticipatory and consummatory anhedonia, and primarily focus on observable behaviors rather than internal experience [3]. Consequently, they fall short in evaluating the internal deficits, which is crucial for fully understanding symptoms like avolition, and asociality. Therefore, the conference advocated for the creation of second-generation rating scales that would better capture both observable behaviors and internal experience associated with negative symptoms. However, psychometric validation of these scales is still in progress, and they are not yet recommended by regulatory agencies, thus limiting their use in clinical trials and settings [21,22,23]. Complementing these traditional methods, remote digital phenotyping, i.e., the collection and analysis of behavioral and physiological data using digital devices (e.g., smartphones and wearable technology), would offer a novel approach to capturing real-time, high-resolution symptom data [24,25].

This review provides a critical overview of both second-generation scales and remote digital phenotyping for assessing negative symptoms in patients with schizophrenia, highlighting future research needs.

## 2. Second-Generation Rating Scales for Negative Symptoms

Following the Consensus Conference, two second-generation rating scales were developed: the Brief Negative Symptom Scale (BNSS) and the Clinical Assessment Interview for Negative Symptoms (CAINS). These instruments were specifically designed to overcome the primary limitations of earlier scales by providing a more refined approach to assessing both the Motivational Deficit and the Expressive Deficit domains of negative symptoms [3]. This advancement allowed for a more comprehensive evaluation, addressing not only observable behaviors but also the internal experiences crucial for understanding deficits like avolition and asociality.

### 2.1. The Brief Negative Symptom Scale (BNSS)

The BNSS [26] is a semi-structured interview tool that assesses 13 items grouped into six subscales, which correspond to the five key domains of negative symptoms—anhedonia, asociality, avolition, blunted affect, and alogia—plus an additional measure of lack of distress. It includes a set of materials such as a manual, a score sheet, and a workbook. The manual explains terminology, provides specific scoring anchors, and includes guidelines for a semi-structured interview with suggested questions. The workbook serves as a quick reference for raters, summarizing the questions and scoring anchors for easier use during assessments. The scale uses higher scores to indicate greater symptom severity across its six subscales; for the distress item, a higher score reflects the absence of negative emotions. A total score is calculated by summing all 13 items, with subscale scores derived by summing the items within each specific subscale. The total score ranges from 0 to 78. The administration of the scale takes about 15 min and focuses on assessing the patient’s experiences and behaviors over the past week [3].

### 2.2. The Clinical Assessment Interview for Negative Symptoms (CAINS)

The CAINS [27,28,29] was developed as part of the Collaboration to Advance Negative Symptom Assessment in Schizophrenia. Its creation followed a data-driven, iterative process that resulted in multiple versions before reaching its final form. The finalized scale comprises 13 items organized into two subscales: the Motivation and Pleasure subscale comprises 9 items evaluating motivation and pleasure across social, occupational, and recreational areas; the Expression subscale comprises 4 items evaluating facial and vocal expression, expressive gestures, and quantity of speech. Each item is rated on a five-point Likert scale from 0 (no impairment) to 4 (severe deficit). Like the BNSS, the CAINS includes a detailed manual and workbook to guide a semi-structured interview. The administration takes about 15–30 min. As for the BNSS, also in this case the assessment period refers to the previous week. While the BNSS separates items that specifically assess internal experiences, the CAINS combines assessments of internal states with observable behaviors into single ratings. A psychometric comparison between the BNSS and CAINS found high agreement for items related to blunted affect and alogia, moderate alignment for avolition and asociality, but lower correspondence for anhedonia items. This discrepancy in assessing anhedonia could be attributed to differences in item definitions and assessment methods. The CAINS focuses on the frequency of pleasurable experiences, with separate items for social, occupational, and recreational pleasures, whereas the BNSS assesses both frequency and intensity of pleasure with a single item covering social, occupational, recreational, and physical pleasure [3].

### 2.3. Advantages and Challenges of Second-Generation Rating Scales

Second-generation rating scales for negative symptoms were specifically designed for both clinical trials and routine clinical practice [3]. Their properties are summarized in Table 1. They offer significant advantages over traditional tools like the PANSS and SANS by providing more comprehensive assessments of negative symptoms. Both scales align with the five domains established by the NIMH Consensus Conference—anhedonia, avolition, asociality, blunted affect, and alogia. Unlike older scales which primarily focus on observable behaviors, BNSS and CAINS stand out by comprehensively assessing both internal experiences and observable behaviors, especially in domains like asociality and avolition [3]. Notably, they distinguish between consummatory and anticipatory components of pleasure, allowing for a more comprehensive evaluation of pleasure deficits [3].

Both the BNSS and CAINS demonstrated solid psychometric properties. Both received a “moderate” grade according to the “COnsensus-based Standards for the selection of health Measurement Instruments” (COSMIN) guidelines, making them valuable tools for assessing negative symptoms in individuals with schizophrenia [22,23]. Both scales showed good internal consistency [22,23], at least for subscale’s scores in the case of the CAINS [23], good interrater reliability, robust hypothesis testing for divergent and convergent validity, especially when compared to older scales such as the SANS [22,23], and good structural validity, especially for the BNSS [22,23]. Their availability in multiple languages and different cultural contexts further enhances their adaptability [3,22,23]. These points highlight the reliability and suitability for clinical use of second-generation rating scales [3,22,23]. In terms of administration time, the BNSS outperforms the CAINS, requiring approximately 15 min compared to the CAINS’ 30 min. This efficiency makes the BNSS particularly advantageous in time-sensitive clinical and research settings [22].

However, despite the advancements made with the development of second-generation rating scales, these tools still present different intrinsic limitations that reduce their effectiveness in fully capturing the complexity of negative symptoms in schizophrenia [11,21,22,23,24,30]. One of the main challenges of these scales is their reliance on structured clinical interviews, which requires comprehensive rater training to ensure accurate administration [24]. This can be especially challenging in resource-limited settings where access to trained clinicians may be scarce. Additionally, both the BNSS and the CAINS rely heavily on retrospective self-reports, which can be affected by patients’ memory, their insight into their symptoms, and their willingness to disclose emotional states and behaviors [24,30]. Such dependence on recall might introduce the risk of recall bias, potentially compromising the accuracy of assessments. Another significant limitation is that, although these scales aim to evaluate both observable behaviors and internal experiences, they are primarily conducted in clinical environments, which may not fully reflect the patients’ everyday experiences [30,31]. Lastly, both scales fail to differentiate primary negative symptoms from secondary ones caused by positive symptoms, depression, or extrapyramidal effects, resulting in diagnostic inconsistencies and treatment challenges [24].

As regards to psychometric properties, one major concern for both scales regards their content validity: the original development of these scales was simply based on the NIMH consensus and did not include cognitive interview studies with patients or clinicians to ensure that the items fully capture the range of negative symptoms as experienced by individuals with schizophrenia and all used terms are comprehensible [22,23]. This gap raises questions about whether the scales comprehensively address all relevant aspects of negative symptoms. According to Weigel et al. (2023), one approach to improving the recommendability of these scales is to retrospectively validate their content validity through focus groups including patients, stakeholders, and caregivers [22].

Moreover, the cross-cultural validity of both scales remains underexplored; while these scales have been translated into several languages, there is limited evidence confirming their validity across diverse cultural contexts [22,23]. This represents an important point that needs to be addressed since language barriers may significantly impact the assessment of negative symptoms of schizophrenia across multiethnic populations [32,33]. Moreover, cultural differences can lead to varied interpretations of negative symptoms, such as avolition and anhedonia, which may not be universally understood [32].

The scale’s test–retest reliability is another area that requires further investigation. For the BNSS, existing studies have primarily relied on Pearson correlations, which are less robust compared to intraclass correlation coefficients (ICCs) recommended by the COSMIN guidelines [22]. The test–retest reliability of the CAINS has also been inconsistently evaluated, with some studies reporting lower than optimal reliability coefficients, which may limit its utility in tracking symptom changes over time [23]. Furthermore, although both scales have been described as tools with a good internal consistency, there are also concerns about this psychometric property. In particular, for the BNSS, most studies reported Cronbach’s alpha only for the overall scale. According to the COSMIN standards, it is advisable to validate the internal consistency for each individual subscale rather than solely relying on the total score [22]. On the other hand, while CAINS showed high alpha coefficients for its individual subscales, there are concerns about the overall internal consistency of the scale. Specifically, the results for internal consistency are often indeterminate due to missing data on structural validity [23]. Therefore, further confirmatory factor analytic studies are needed in order to establish the structure validity of this scale. Additionally, the convergent validity of the CAINS has predominantly been assessed in comparison to first-generation rating scales. Therefore, there is a need for further studies comparing it to second-generation scales like the BNSS to better understand its effectiveness [23]. Lastly, another point of critique involves the inclusion of a distress item in the BNSS scale, which does not align with the original five domains defined by the NIMH-MATRICS Consensus [3,22]. This item has shown lower reliability scores and may not fit well with the other domains, raising concerns about its relevance within the scale.

In conclusion, the psychometric validation of the BNSS and the CAINS has demonstrated notable improvements in reliability and validity, and they are therefore rating tools with the potential to be recommended for clinical use. However, key challenges remain, including the need for broader cross-cultural validations and retrospective analyses of content validity through cognitive interview studies with patients and/or clinicians. Addressing these gaps will be essential to achieving recognition by regulatory authorities. Future efforts should focus on differentiating primary negative symptoms from secondary ones, standardizing assessment methodologies across diverse populations, and demonstrating the utility of these scales in multicenter clinical trials.

## 3. Remote Digital Phenotyping for Negative Symptoms

Despite the advancements made with the development of second-generation rating scales such as the BNSS and the CAINS, as described in Section 2.3, these tools still present several intrinsic limitations that reduce their effectiveness in fully capturing the complexity of negative symptoms in schizophrenia [3,21,22,23,24,30].

Within this context, in February 2023, the Negative Symptoms Work Group of the International Society of CNS Clinical Trials and Methodology (ISCTM) recognized the limitations of current clinician-administered rating scales and agreed on the need for a new multimodal tool to evaluate negative symptoms [30]. The experts highlighted the potential of remote monitoring to address many of these shortcomings, though no remote assessment of negative symptoms has yet been validated as a primary outcome measure for clinical trials [30].

Remote digital phenotyping leverages digital technologies—such as smartphones, smartwatches, heart rate monitors, and other wearable devices—to collect both actively solicited and passively measured data on an individual’s behavior and physiological states in real time [34]. This real-time data collection can reveal subtle symptom changes that might not be apparent during clinician-administered assessments [30,35,36]. By continuously monitoring behaviors such as physical activity, social interaction, and emotional expression, remote digital phenotyping could offer a more ecologically valid and comprehensive understanding of negative symptoms, capturing not just the severity of symptoms but also their impact on patient’s daily functioning. This holistic insight can ultimately guide more personalized and effective treatment strategies [31,37].

Different methods have been used as remote digital phenotyping for the evaluation of negative symptoms, such as the Ecological Momentary Assessment (EMA), the analysis of facial and vocal expression, the Global Positioning System (GPS), and the actigraphy [24,31]. One of the key strengths of remote digital phenotyping is its ability to reduce recall bias and increase objectivity compared to traditional assessment methods. By leveraging automated analyses of behavioral patterns, facial expressions, and speech, it enables continuous, real-time monitoring of behaviors and physiological indicators, providing a detailed record of symptom fluctuations and changes over time [30].

While remote digital phenotyping offers substantial promise, it is important to emphasize that these tools are not intended to replace clinician-administered measures. Instead, they should be viewed as complementary, providing additional insights and improving the precision of symptom assessment.

### 3.1. Ecological Momentary Assessment (EMA)

The Ecological Momentary Assessment (EMA) is one of the most widely used methodologies within the framework of remote digital phenotyping [31,38]. It leverages mobile devices to prompt participants to report on their current emotional states, activities, and social interactions in real time, thereby collecting data that reflect their everyday experiences. Data is collected through surveys, often personalized on the basis of various contexts, such as time of the day, emotional state, and localization [31,38]. Unlike traditional retrospective assessments, EMA reduces recall bias and captures moment-to-moment fluctuations in symptoms, which can be missed during scheduled clinical interviews. In this way, the use of EMA could significantly reduce data collection time in psychiatric research [39], and its integration within traditional assessment methods can significantly enhance the predictive validity of psychiatric diagnoses and clinical outcomes [40]. EMA requires participants to respond to frequent prompts (maximum reported in the literature 30 surveys per day) for a time range of evaluation that generally is set between 1 and 2 weeks (maximum reported in the literature 30 days) [38]. EMA can be administered either at predetermined times of the day (fixed time) or through notifications distributed at random times throughout the day (random time) [38,41]. Studies comparing these approaches in individuals with schizophrenia highlight their respective strengths and weaknesses. The random method offers greater flexibility and is less prone to retrospective biases, which can result in higher patient engagement and more ecologically valid data [38,41]. Conversely, the fixed method allows for more structured data collection and facilitates a more straightforward interpretation of data trends over time, but it may fail to capture critical symptom variability and contextual factors influencing psychosis [38,41]. Currently, there is no definitive evidence favoring one method over the other, specifically for patients with schizophrenia. A blended approach combining fixed and random sampling has been proposed as a potential optimization strategy, aiming to balance flexibility, ecological validity (the extent to which assessment methods reflect patients’ real-world experiences and daily functioning), and the consistency of data collection [38,41].

EMA has proven to be a feasible and an effective method for patients with schizophrenia, showing a mean completion rate of 67.15% [38], demonstrating that patients are willing and able to engage with this method despite their cognitive and motivational impairment. Different factors, such as incentives to patients, the time interval between subsequent evaluations, the number of prompts, intelligence quotient (IQ), age, severity of symptoms, and, importantly, environmental and contextual factors, might impact the completion rate. However, in the meta-analysis by Bell et al. 2024 [38], the authors reported that variables such as age, pre-morbid IQ, working memory, and severity of symptoms, were not significantly related to completion rates [38]. Further studies are needed to clarify the role of incentives, EMA methodologies, and environmental and contextual factors on the adherence rates. For instance, some studies proposed and tested different strategies to effectively increase patient motivation prior to starting a survey. These strategies focus on enhancing communication using tailored communication methods, providing incentives, and utilizing technology to engage patients more effectively [42].

As regard to negative symptoms, EMA has been used to assess symptoms belonging to the Motivational Deficit Domain, such as anhedonia, avolition, and asociality, by evaluating both the internal experiences and the behavior [43]. In particular, the internal experience is evaluated by asking participants how much he/she is motivated to be engaged in goal-directed, social activities; the behavioral component is measured via binary checklist items completed for each survey regarding whether participants were engaged in social, recreational, or goal-directed activities.

The first study that employed EMA to evaluate motivational deficits in subjects with schizophrenia was that of Moran et al. 2017 [44]. In this study, 34 subjects with schizophrenia or schizoaffective disorder, were asked to respond to an EMA questionnaire with 9 questions regarding their current activities (i.e., work/school; TV/music/reading/exercise; chores; social activity; sleeping; eating; nothing in particular) and their motivation and pleasure in these activities on a 5-point scale from “not at all” to “extremely”. The authors found that clinician-rated measures of negative symptoms assessed with the CAINS were significantly related to negative symptoms assessed via EMA. This relationship was moderated by working memory, such that there was a stronger relationship among patients with better working memory functions, thus suggesting that working memory impairments frequently seen in schizophrenia may affect recall of symptoms [44]. The finding of a good convergent validity of the EMA measures with traditional measures has been also replicated in an independent study using the BNSS [45]. In a subsequent work conducted by the same group of Moran et al. [46], the authors used EMA procedures to investigate goal-directed behaviors in subjects with schizophrenia. Also in this case there was a correlation between EMA measures and the CAINS MAP scores [46].

Additionally, EMA has been successfully used to evaluate social interactions. For instance, Kasanova et al. (2018) found that patients with schizophrenia spent significantly less time in structured social contexts compared to healthy controls. Interestingly, while patients reported intact hedonic experiences during social interactions, they exhibited less frequent social engagements, especially in structured social contexts, which correlated with higher ratings of avolition [47]. However, it is worth noting that, in this study, the assessment of avolition was not in line with the current conceptualization [3], as it included PANSS emotional withdrawal, passive social withdrawal, and active social avoidance.

Recent research has further extended the use of EMA in assessing negative symptoms, particularly focusing on the role of emotional experiences and their impact on motivation. In this context, Bartolomeo et al. (2023) [48] explored the positivity offset theory in schizophrenia, which suggests that patients may experience reduced positive emotions in low-arousal contexts, thereby affecting approach behaviors and motivation. In this study, participants with schizophrenia and healthy controls were assessed using a combination of active EMA surveys evaluating the momentary emotional experiences and arousal, as well as anhedonia, avolition, and asociality, alongside passive data collection methods (e.g., geolocation, which will be explained in Section 3.3) over one week. The results showed that individuals with schizophrenia had a diminished positivity offset, which correlated with more severe anhedonia and avolition, assessed through digital phenotyping and BNSS. These findings indicated that emotional experience abnormalities may underlie motivational deficits in schizophrenia [48]. Importantly, the findings highlight the value of combining traditional clinical rating scales with both active and passive digital phenotyping methods to obtain a more comprehensive picture—not only of patients’ symptomatology but also of the underlying behavioral mechanisms driving negative symptoms. This integrated approach can enhance clinicians’ understanding of both the presentation and the behavioral foundations of negative symptoms, ultimately leading to more precise assessments and targeted treatment strategies.

### 3.2. Analysis of Facial and Vocal Expression

The analysis of facial and vocal expression has been used to evaluate symptoms belonging to the Expressive Deficit domain, such as blunted affect and alogia [49,50]. In the study conducted by Cohen and colleagues (2020), the participants were asked to complete surveys on their recent social interactions, activities, and locations, alongside recording short videos “selfie” where they described the events of the past hour in detail. To analyze the facial and vocal features, the researchers utilized tools like FaceReader for facial expressions and the Computerized Assessment of Affect from Natural Speech for vocal analysis. The completion rate for the video recording was low (23% for patients and 27% for healthy controls). This method showed convergent validity with clinician-rated scales like the BNSS, suggesting that objective digital assessments could align with traditional clinical evaluations of negative symptoms. However, the study revealed some unexpected findings. For example, while pause length, which is typically associated with alogia, was correlated with several clinician-rated negative symptoms, it did not specifically correlate with alogia itself. Conversely, the number of utterances was found to be linked primarily to alogia. Similarly, blunted affect, as rated by clinicians, did not correlate with less varied vocal intonation or emphasis but was instead associated with increasing negatively and positively valenced facial expressions [49]. The authors explained that these discrepancies highlight how traditional clinical assessments may fail to fully capture the complexity of behavioral features measured in real-time contexts [49,50]. Certainly, these discrepancies warrant further research on the different aspects of verbal and non-verbal communication, evaluated through either automated analysis or clinical assessments [51,52]. Furthermore, the study found that the temporal stability for the vocal production and the facial expression features was lower compared to clinician-administered scales, possibly suggesting that remote digital phenotyping can be more sensitive to detecting changes over time, thus capturing fluctuations that may not be apparent during structured clinical evaluations [49,50]. Finally, vocal and facial features made important incremental contribution to both BNSS ratings and EMA-based measures for predicting work and social activities [49].

Another study used the measurement of the head movement recorded through remote smartphone-based assessments, in order to assess motor functions in 18 subjects with schizophrenia. The authors found that subjects with schizophrenia, as compared to healthy controls, showed a reduced rate of head movement, which was a predictor of the schizophrenia diagnosis and was negatively correlated with PANSS scores of blunted affect, emotional withdrawal, and poor rapport [53]. These findings suggest that smartphone-based tools can effectively assess motor dysfunctions and negative symptom severity in schizophrenia, offering a valuable complement to traditional assessment scales.

### 3.3. Geolocation

Geolocation uses GPS coordinates to monitor a person’s location at set intervals or when they move beyond a specified distance. By tracking movement patterns, geolocation can provide valuable insights into factors like the time spent in specific locations, distances travelled, and overall mobility levels. This technique has been shown to be feasible for individuals with schizophrenia, demonstrating strong correlations with self-reported EMA data and clinical assessments [43,54]. However, while GPS tracking has generally been well-received by clinically stable patients [43], it may present challenges for individuals experiencing more severe psychotic symptoms [31]. Encouragingly, adherence to GPS monitoring has been reported to exceed 75% and was not influenced by potential confounders such as age, education, gender, and living situation [43]. However, further research is needed in order to confirm these findings.

Research using GPS monitoring may serve as a valuable tool for objectively assessing negative symptoms [43,54]. For instance, patients with schizophrenia exhibited lower average distances travelled and shorter distances from home [43,54], a higher proportion of time spent at home compared to healthy controls [54]. Interestingly, reduced travel distance and proximity to home were associated with increased severity of negative symptoms belonging to the CAINS MAP scale [54]. Furthermore, geolocation has demonstrated convergent validity by small to medium correlations with EMA indices and BNSS scores of avolition, asociality, and anhedonia, in particular with the behavioral evaluation [43]. It showed also good discriminant validity, as demonstrated by low correlations with positive symptoms, anxiety and depression, good internal consistency, and moderate stability over time [43]. Interestingly, it seems that GPS mobility is quite a specific marker of negative symptoms since no correlation has been reported with functioning, positive symptoms, depression, or neurocognition [43].

### 3.4. Actigraphy

Actigraphy, a method that utilizes wearable devices like wristbands or smartwatches, has become an effective tool for objectively measuring physical activity and circadian rhythms in individuals with schizophrenia. By continuously recording movements, actigraphy provides a non-invasive means of collecting real-time data on patients’ daily routines, sleep patterns, and overall activity levels [31]. This technology is particularly useful for monitoring motor abnormalities, which are common in schizophrenia [55,56], and it has also been used for the evaluation of negative symptom severity [56].

In the study of Umbricht et al. (2020) [56], patients with schizophrenia wore a wrist-worn actigraphy device over a 15-week period, which allowed researchers to continuously assess movement patterns and correlate these with clinical ratings of negative symptoms assessed with the BNSS [56]. Findings from this study indicated that reduced activity levels were associated with greater severity in negative symptoms, specifically within the domains of the Expressive Deficits.

### 3.5. Advantages and Challenges of Remote Digital Phenotyping

As highlighted in the previous paragraphs, the use of remote digital phenotyping in schizophrenia offers significant advantages, especially when combining both active and passive assessments with traditional methods [31,48]. One of the primary benefits of this approach is its ability to collect continuous, real-time data, providing valuable insights into negative symptoms as they manifest in real-world settings. The Ecological Momentary Assessment (EMA) allows for frequent surveys that capture both internal experiences and behaviors, while actigraphy and GPS track physical movement and activity patterns, offering objective data on behaviors often overlooked in clinical assessments. Additionally, facial and vocal analysis through wearable devices or smartphone cameras provides high-resolution data on emotional expressions and speech patterns, helping to identify subtle changes in symptoms such as blunted affect and alogia. By reducing the subjectivity inherent in clinician ratings, objective data collection may offer a more accurate and individualized understanding of a patient’s condition. However, further research is needed to explore the underlying causes of discrepancies between automated analyses and clinical assessments [31]. The accessibility and scalability of smartphones and wearables make this approach particularly beneficial for patients with limited access to healthcare facilities. Finally, the longitudinal nature of digital phenotyping might also enable continuous assessment, offering insights over time that can inform ongoing care [21].

However, different challenges of these techniques have to be considered [31].

For instance, the use of mobile or electronic devices can potentially lead to increased suspicion and paranoia in subjects with psychosis. However, the evidence on this topic remains indirect. Specifically, these digital instruments, which capture real-time data on psychotic symptoms, may heighten patients’ awareness of their symptoms, potentially exacerbating feelings of paranoia [57]. Future studies are warranted to investigate these potential risks directly and to develop strategies that minimize adverse effects while ensuring the safety and effectiveness of digital tools for all patients.

Furthermore, active digital phenotyping, such as EMA, often involves long surveys and high participant burden, which may decrease adherence rates. In turn, low adherence rates can introduce “moment selection bias”, as participants may avoid EMA assessments during situations they find inappropriate or inconvenient, such as noisy or distracting environments [58,59,60]. This is particularly relevant for patients with cognitive impairments, who may struggle more in such contexts. Missing data resulting from these contextual factors poses a significant threat to the ecological validity and generalizability of findings, underscoring the need for further investigation into the magnitude and impact of selection effects [58]. Additionally, technical issues, such as battery life or software malfunctions in devices, might further compromise data collection. All these aspects complicate the interpretation of results, and future studies should include thorough analyses of factors causing participants to miss assessments. While many studies report good adherence rates, it is important to note that some participants received compensation, which could also influence results [31].

Ensuring patient privacy and data security in the context of real-time data collection is fundamental. Achieving this requires balancing regulatory compliance with patient autonomy and ensuring transparency regarding data use and storage. This could be addressed by including robust encryption protocols, clear consent processes, and adherence to regulatory frameworks and by implementing novel approaches (e.g., federated learning) to enhance data protection while maintaining analytic rigor.

Furthermore, the continuous collection of sensitive personal data poses questions of professional responsibility and the clinician’s duty and right to intervene, might he/she receive worrying signals [61,62]. For instance, digital phenotyping tools may enhance the detection of emergencies by analyzing real-time patient responses and behavioral patterns. Therefore, they can serve as effective intermediaries by identifying critical situations (e.g., high suicide risk, psychosis exacerbation) and automatically alerting healthcare providers. However, while digital phenotyping has shown promise in suicide prevention within non-psychotic populations, to date, it has not been used to predict suicidal ideation or behavior in individuals with schizophrenia. This represents a critical gap in the literature and an important avenue for future research.

Another limitation is related to the small sample sizes of existing studies, sometimes lacking appropriate control groups, which restricts the robustness of the findings. Furthermore, the majority of studies have focused on clinically stable patients, thus limiting the generalizability of the results to patients with more severe symptoms or those in different stages of the illness. Moreover, collecting data from healthy subjects and integrating it into analyses is crucial to contextualize findings better. Large-scale studies in diverse patient populations are needed to determine the broader applicability of remote digital phenotyping, as well as to validate the feasibility, acceptance, and effectiveness of these methods. Improving patient acceptance of digital phenotyping technologies requires addressing concerns related to usability, privacy, and perceived benefits. Educational initiatives to increase awareness and understanding of these technologies, combined with developing user-friendly interfaces tailored to patient needs, can significantly enhance engagement. Moreover, ensuring accessibility in resource-limited areas calls for the adoption of low-cost solutions and the involvement of community healthcare workers for effective implementation. Collaborations between technology developers, clinicians, policymakers, and multiple stakeholders are crucial to overcoming these barriers and ensuring equitable access to these technologies [21,31]. This process should serve also to assure data protection, ensuring the sustainability of available apps, collecting normative data, and developing validated measures of negative symptoms in real-world settings.

## 4. A Complementary Tool in Remote Digital Phenotyping: The Computerized Adaptive Testing (CAT)-Psychosis

An additional example of innovative technology relevant to the field of remote digital assessment is the CAT-Psychosis, a computerized adaptive testing system developed by Guinart et al. (2021) [63]. This tool leverages item response theory to dynamically adjust the questions based on the patient’s previous responses, thereby reducing assessment time to an average of 5 min for clinician-administered versions and just over 1 min for self-administered versions. Importantly, the CAT-Psychosis has been designed for use on devices such as smartphones, tablets, and computers, enabling its application in remote settings [63].

While the CAT-Psychosis exemplifies how technology can improve the efficiency of symptom assessment, it is important to note that this tool is not specifically designed to assess negative symptoms. Instead, it evaluates a broader range of psychopathological domains, including also positive and disorganized symptoms. Additionally, for the assessment of negative symptoms, the CAT-Psychosis relies on the SANS [63], which, as already mentioned in this paper is a first-generation rating scale for negative symptoms developed before the MATRICS Consensus Conference. Nevertheless, the CAT-Psychosis offers a complementary perspective, demonstrating the feasibility of rapid, remote assessments that could be adapted or integrated into future developments specifically targeting negative symptoms.

## 5. Conclusions and Future Directions

In conclusion, the assessment of negative symptoms in schizophrenia has made significant progress with the development of second-generation rating scales, such as the Brief Negative Symptom Scale and the Clinical Assessment Interview for Negative Symptoms. While these tools address some of the limitations of first-generation rating scales, their psychometric validation is still in progress. As such, they are not yet widely endorsed by regulatory agencies, which limits their application in clinical settings and trials.

Remote digital phenotyping represents a promising complement to traditional methods by leveraging smartphones and wearable devices to collect real-time, high-resolution data on symptoms. This approach presents distinct advantages, such as continuous monitoring, objective data collection, and the ability to detect subtle changes in negative symptoms in real-life contexts. While these digital methods are promising, they are still in the early stages of validation and are not yet ready for widespread use in clinical practice or clinical trials. One of the main challenges is ensuring their ecological validity—how well they capture real-world symptom changes—and their integration into existing clinical workflows. Additionally, issues such as patient acceptance and adherence, data privacy and security, and technological accessibility need to be addressed before these tools can be routinely used in diverse clinical settings. Bridging this gap will require large-scale studies to validate these tools across diverse patient populations, with a particular focus on their feasibility and clinical utility.

Finally, it is essential to emphasize that remote digital phenotyping methods are not intended to replace clinician-administered assessments. Rather, they should be seen as complementary tools that provide additional insight, enhancing the precision of symptom evaluation. For example, technologies like CAT-Psychosis [63] could potentially be adapted to focus specifically on negative symptoms. Experts and different stakeholders in the field should collaborate to initiate the development of a new multimodal instrument specifically designed for assessing negative symptoms [30], by integrating traditional assessment methods with digital phenotyping. This new tool should incorporate core functionalities such as real-time symptom monitoring, objective behavior tracking, and patient-reported outcomes. Furthermore, combining EMA with passive data collection methods (e.g., actigraphy, geolocation) could enhance ecological validity while maintaining clinical relevance. Such innovative approaches could redefine how negative symptoms are assessed and managed in diverse healthcare settings, paving the way for more precise, individualized interventions. While this process may present challenges, it offers the opportunity to integrate digital phenotyping into routine clinical practice, ultimately leading to more precise, personalized, and timely interventions for negative symptoms in individuals with schizophrenia [30,35,36].

## Figures and Tables

**Table 1:** Properties of the Brief Negative Symptom Scale (BNSS) and the Clinical Assessment Interview for Negative Symptoms (CAINS).

	BNSS	CAINS
Assessed Domains	Anhedonia, Asociality, Avolition, Blunted Affect, Alogia, Lack of Distress	Motivation and Pleasure (social, occupational, recreational) and Expression (facial, vocal, gestural, speech)
Time for Administration	~15 min	15–30 min
Investigated Period	Past week	Past week
Psychometric Properties	Good internal consistency (total scale); good interrater reliability; robust hypothesis testing for divergent and convergent validity; moderate test–retest reliability	Good internal consistency (subscales); good interrater reliability; robust hypothesis testing for divergent and convergent validity; moderate test–retest reliability
Strengths	Distinction between consummatory and anticipatory pleasure; shorter administration time	Focus on real-life motivational experiences; inclusion of broad social, occupational, and recreational contexts
Limitations	Requires experienced raters; lack of robust cross-cultural validation; retrospective self-reports; primarily conducted in clinical environments; low content validity testing	Requires experienced raters; lack of robust cross-cultural validation; retrospective self-reports; primarily conducted in clinical environments; low content validity testing
